# True superior gluteal artery aneurysm with neurovascular compromise of the lower limb: a case report and review of the therapeutic options

**DOI:** 10.11604/pamj.2018.30.135.12509

**Published:** 2018-06-14

**Authors:** Tunde Nureini Oyebanji, Ismail Mohammed Inuwa, Jameel Ismail Ahmad

**Affiliations:** 1 Department of Surgery, Aminu Kano Teaching Hospital, PMB 3452, Zaria Road, Kano, Nigeria; 2 College of Health Sciences, Bayero University of Kano and Department of Surgery, Aminu Kano Teaching Hospital, PMB 3452, Zaria Road, Kano, Nigeria

**Keywords:** Superior gluteal artery, aneurysm, surgery

## Abstract

Few cases of true superior gluteal artery (SGA) aneurysms have been described in the English-language literature. This is the twenty-second reported case. SGA aneurysms can pose diagnostic problems, specifically when they are non-pulsatile and also therapeutic challenges when they are large. Although more aneurysms are being subjected to endovascular therapies, SGA aneurysmectomy or aneurysmorrhaphy still remain valid therapeutic options, especially in resource-poor settings. Surgery provides quick symptom resolution and still is the only means by which tissue for definitive histological diagnosis can be obtained.

## Introduction

A true aneurysm of the superior gluteal artery (SGA) is a rare clinical condition with only twenty-one previously reported cases [1] in the English-language literature. Complications include compressive symptoms, rupture with exanguination, insitu thrombosis and embolic phenomena causing limb ischaemia. We present the first case of true SGA aneurysm from our centre and aim to review the literature on the current therapeutic options.

## Patient and observation

**Figure 1:**
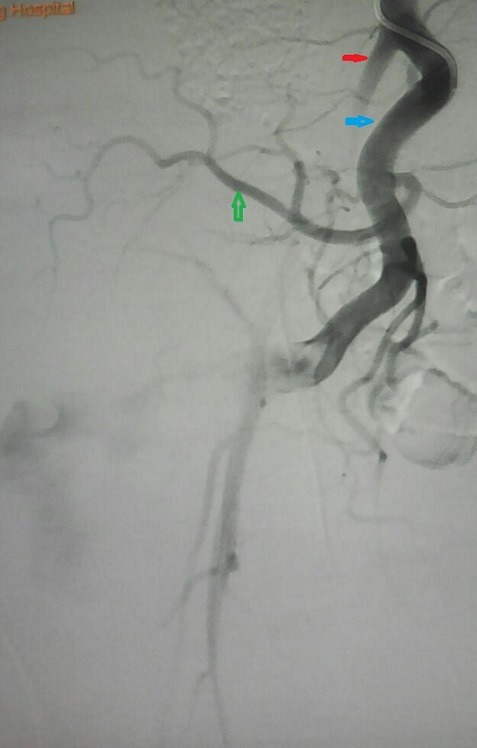
Femoral angiogram: blue arrow = internal iliac artery, red arrow = external iliac artery, green arrow = lateral sacral artery

**Figure 2:**
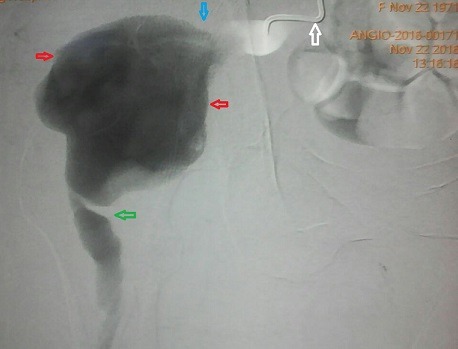
Red arrows = superior gluteal aneurysm, blue arrow: proximal neck, green arrow = distal neck, white arrow = angio catheter

**Figure 3:**
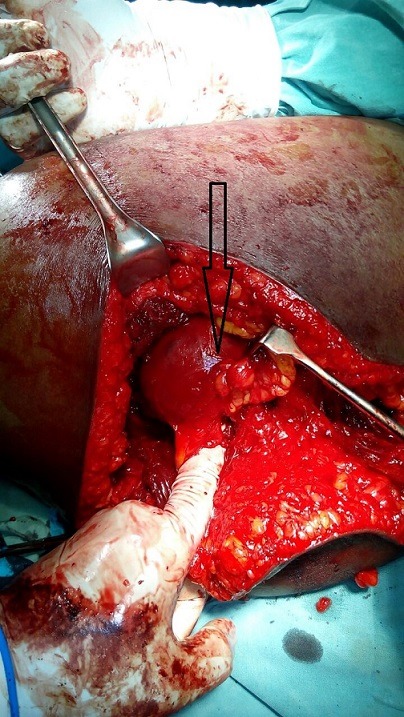
Mobilization of the aneurysm via the gluteal incision; black arrow = aneurysm

**Figure 4:**
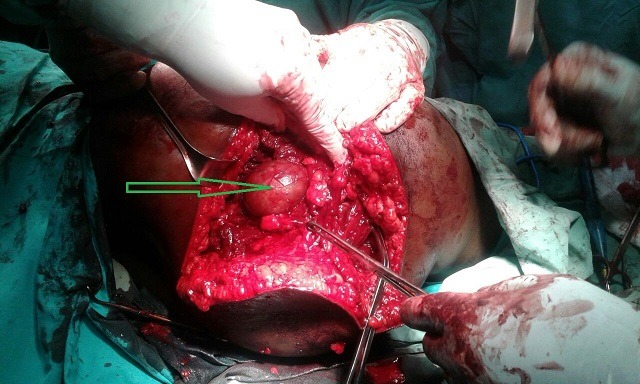
Green arrow showing fully mobilized aneurysm

**Figure 5:**
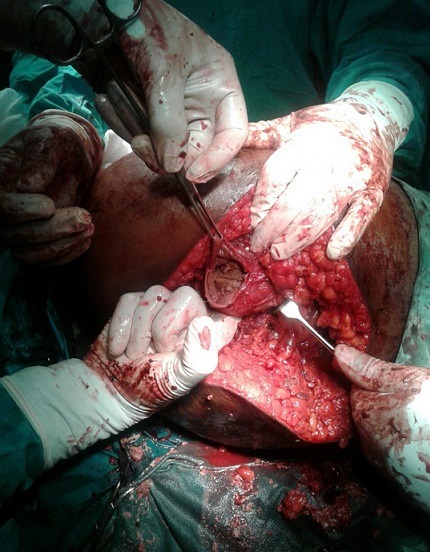
Incision of the aneurysm and evacuation of thrombus

**Figure 6:**
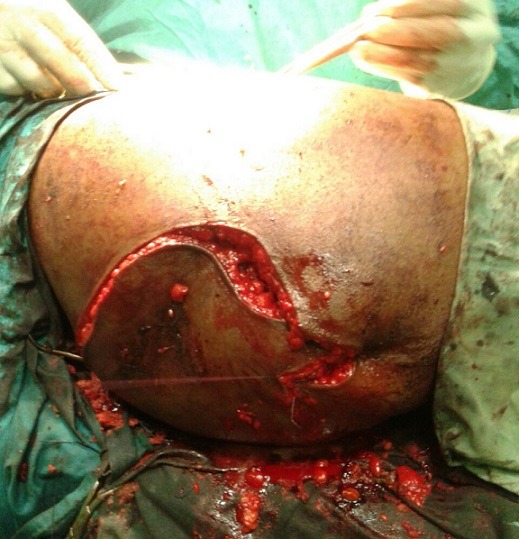
Incision being closed

A 45-year-old woman presented to the outpatient department with a 2-year history of right gluteal swelling which was initially painless but later painful as it increased in size. The pain radiated down the right thigh and knee. She later developed paraesthesia and darkening of the right big toe and subsequent ulceration. She never complained of intermittent claudication or rest pain. She was a known hypertensive. There was no history of cigarette smoking, dyslipidaemia or diabetes mellitus. She never experienced any trauma to the buttock or any other part of the body and neither was she treated for any chronic infectious or inflammatory condition. There was no history suggestive of a connective tissue disease. The musculoskeletal system examination revealed a pulsatile mass of about 10 x 8cm in the right gluteal region with an accompanying bruit. There were trophic changes over the right foot and ankle with evidence of a healed ulcer over the right big toe. The foot was warm to touch but there was sensory loss involving L4-S1 dermatomes over it. The gross power in the right lower limb was 2/5 based on the MRC scale. The femoral and popliteal pulses were bilaterally palpable. The right dorsalis pedis and posterior tibial pulses however were not appreciable. She had a blood pressure of 160/90mmHg. Femoral angiography revealed a fusiform 12 x 8cm aneurysm of the superior gluteal artery (SGA) with a wide neck which would prove difficult to embolize with the available coils (Figure 1, Figure 2). Aneurysmectomy of the SGA was planned and done. Proximal control of the Internal Iliac Artery was obtained via an extraperitoneal groin incision before a gluteal incision was made to mobilize the gluteus maximus muscle. A true fusiform 12 x 8cm SGA aneurysm was found under the muscle which was firmly adherent to and stretching the sciatic nerve (Figure 3, Figure 4). It contained a huge thrombus (Figure 5). The nerve was separated from the aneurysm by blunt and sharp dissection and the aneurysmectomy was done. The wound was closed in layers (Figure 6). Early postoperative mobilization and physiotherapy were encouraged and she shortly regained the use of the affected limb. She developed a surgical site infection which prolonged her hospital stay. This was managed with daily dressing and culture and sensitivity based antibiotic therapy until she was discharged. Histological analysis of the specimen revealed several congested and dilated blood vessels containing red blood cells with other areas showing necrosis and inflammatory cells.

## Discussion

The SGA is the largest branch of the posterior division of the Internal Iliac artery. It exits the greater sciatic foramen superior to the piriformis. The sciatic nerve exits the same foramen below the piriformis. Most aneurysms of the gluteal artery are pseudo-aneurysms caused by blunt or penetrating trauma from falls on the buttock [2], pelvic surgeries, pelvic fractures, intramuscular injections etc. True aneurysms are rare with only twenty-one previously reported cases [1]. The commonest true SGA aneurysms are of mycotic origin secondary to intravenous drug abuse [3] or infective endocarditis [4,5]. True atherosclerotic SGA aneurysms have been described, some from histological examination of resected tissue [4] and others from clinical and morphological features of the aneurysm [1]. Immunosuppression has been reported, albeit in the inferior gluteal artery, to be a risk factor for non-typhi Salmonella induced gluteal aneurysm [6]. Polyarteritis nodosa (PAN) was reported as a cause by Gostigian et al [7]. PAN is characterized by the presence of inflammatory reactions of blood vessels of medium or small caliber that lead to necrosis and destruction of the walls of vessels [8]. The American College of Rheumatology have 10 criteria for the diagnosis of PAN [9]. Katz et al [10] reported a case of SGA aneurysm due to the rare intimomedial degeneration involving the circumferential deposition of large amounts of mucoid material within the intima and media of the arterial wall, causing weakening that results in aneurysm formation of the involved segment. The aneurysm causes compression of the sciatic nerve giving rise to the characteristic pain that arises in the ipsilateral buttock and radiates down the thigh and knee. Symptoms of limb ischaemia can arise due to thrombosis or distal embolism [1] just like in our patient who had diminished ipsilateral dorsalis pedis pulse and recurrent pedal arterial ulcers. The aneurysms are expected to be pulsatile when examined. However, cases have been described of non-pulsatile gluteal artery pseudo-aneurysms which have been aspirated [11] or incised [12] with untoward consequences. These illustrations mandate the consideration of gluteal artery aneurysms as differential diagnosis of gluteal swellings which include lesions like gluteal abscesses (cold or pyogenic), lipomas, soft tissue sarcomas, sciatic hernia, Echinococcal cysts, cystic hygroma etc [11]. Diagnostic imaging options include duplex ultrasonography, computerized tomographic angiography (CTA), magnetic resonance angiography (MRA) and catheter-based angiography. CTA is the most effective diagnostic tool, providing exact information regarding the presence, location, diameter, and extension of the aneurysm [13]. CT with three-dimensional (3D) reconstruction evaluates the exact length of the proximal and distal necks because these measurements have the greatest influence on the choice of therapeutic option [13]. MRA is beneficial in patients with renal failure and in pregnancy because it utilizes potentially less nephrotoxic contrast agent and doesn't use ionizing radiation. MR imaging can also depict vascular flow without injection of contrast material [14].

**Surgical treatment**: Currently, the two-staged extraperitoneal approach is favoured [11,12,15]. Transperitoneal approaches have also been described but the former has the advantage of avoiding entry into the abdomen hence precluding potential intra-abdominal abscesses and other complications. Aneurysmectomy or aneurysmorrhaphy [4,11] is the goal. The sciatic nerve forms part of the wall of the sac and should be identified and separated from the sac. True gluteal aneurysms that are solely extra pelvic have been managed from a gluteal approach alone [12,15]. Surgery enables early resolution of symptoms caused by compression or displacement of adjacent structures [16]. In addition, it is only surgery that provides specimen for histological analysis. Surgery, however, may be fraught with technical difficulties. Mortality rates between 0-10% have been quoted for surgical treatment of iliac aneurysms [17]. Hospital stay is increased particularly when surgical site infection occurs. Blood transfusion may be required to correct anaemia when there is significant blood loss.

**Endovascular therapy**: More and more lesions are being subjected to endovascular therapy. Endovascular therapy decreases hospital stay and the chances of surgical site infection. The requirement for blood transfusion is eliminated. There may be, however, delayed symptom resolution and recurrence of the aneurysm. It is also seldom available or affordable in resource-poor settings. Endovascular treatment is recommended for aneurysms that are less than 50 mm in diameter or are asymptomatic, especially in high-risk patients [17]. Other authors have recorded successes with larger aneurysms [18,19]. Therapeutic endovascular methods include coil embolization, thrombin injection and stent-graft placement. Care has to be taken to identify and embolize all afferent and efferent vessels of the aneurysm if possible, to avoid retrograde flow from collateral arteries [3] during coil embolization. Literatures have reported few cases of coil dislodgement and migration [20], particularly if smaller coils are used. This informed our decision to opt for surgery. Thrombin has been injected under ultrasound guidance (UGTI) into pseudoaneurysms [3] to cause thrombosis. UGTI may be more successful in acute superficial aneurysms with narrow necks and not in chronic and large sized aneurysms which may require larger amount of thrombin. In rare cases, thrombin reflux into the circulation with undesired serious thrombosis has been reported [8]. Since most true SGA aneurysms are of mycotic origin, stent-graft placement may not be a first-line option because of the high risk of stent-graft infection [13]. On the other hand, in poor surgical candidates, stent-graft placement may be used to gain time to improve the patient's condition prior to open surgery and, in certain patients, may even become the definitive treatment [13].

## Conclusion

A true SGA aneurysm is a rare condition but there is need for a heightened index of suspicion for its diagnosis. Surgical repair still remains relevant despite the advent of less invasive techniques especially in developing countries.

## Competing interests

The authors declare no competing interests.
